# NMR and Mutational Identification of the Collagen-Binding Site of the Chaperone Hsp47

**DOI:** 10.1371/journal.pone.0045930

**Published:** 2012-09-25

**Authors:** Maho Yagi-Utsumi, Sumi Yoshikawa, Yoshiki Yamaguchi, Yohei Nishi, Eiji Kurimoto, Yoshihito Ishida, Takayuki Homma, Jun Hoseki, Yoshimi Nishikawa, Takaki Koide, Kazuhiro Nagata, Koichi Kato

**Affiliations:** 1 Institute for Molecular Science and Okazaki Institute for Integrative Bioscience, National Institutes of Natural Sciences, Okazaki, Japan; 2 Graduate School of Pharmaceutical Sciences, Nagoya City University, Nagoya, Japan; 3 Systems Glycobiology Research Group, Chemical Biology Department, Advanced Science Institute, RIKEN, Wako, Japan; 4 Faculty of Pharmacy, Meijo University, Nagoya, Japan; 5 Institute for Frontier Medical Sciences, Kyoto University, Kyoto, Japan; 6 Laboratory of Molecular and Cellular Biology, Faculty of Life Sciences, Kyoto Sangyo University, Kyoto, Japan; 7 School of Advanced Science and Engineering, Waseda University, Tokyo, Japan; MRC National Institute for Medical Research, United Kingdom

## Abstract

Heat shock protein 47 (Hsp47) acts as a client-specific chaperone for collagen and plays a vital role in collagen maturation and the consequent embryonic development. In addition, this protein can be a potential target for the treatment of fibrosis. Despite its physiological and pathological importance, little is currently known about the collagen-binding mode of Hsp47 from a structural aspect. Here, we describe an NMR study that was conducted to identify the collagen-binding site of Hsp47. We used chicken Hsp47, which has higher solubility than its human counterpart, and applied a selective ^15^N-labeling method targeting its tryptophan and histidine residues. Spectral assignments were made based on site-directed mutagenesis of the individual residues. By inspecting the spectral changes that were observed upon interaction with a trimeric collagen peptide and the mutational data, we successfully mapped the collagen-binding site in the B/C β-barrel domain and a nearby loop in a 3D-homology model based upon a serpin fold. This conclusion was confirmed by mutational analysis. Our findings provide a molecular basis for the design of compounds that target the interaction between Hsp47 and procollagen as therapeutics for fibrotic diseases.

## Introduction

Heat shock protein 47 (Hsp47) is a 47-kDa protein that operates as a client-specific chaperone for collagen [1]. Growing evidence indicates that this protein plays an important role in the bundle formation of procollagen and the prevention of unfavorable aggregation in the early secretory pathway of collagen [1], [2]. Hsp47 binds and thereby stabilizes the triple helix of procollagen in the pH-neutral endoplasmic reticulum (ER), subsequently dissociates under low pH conditions of the *cis*-Golgi or ER-Golgi intermediate compartment, and recycles back to the ER [3]. A gene ablation study demonstrated that Hsp47 is essential for the maturation of collagen and subsequent embryonic development [4], [5]. However, a high level of Hsp47 expression has been shown to be a risk factor for fibrosis, which is an intractable disease characterized by the abnormal accumulation of collagen in liver and kidney [6], [7], [8]. Hence, Hsp47 is a potential therapeutic target for the treatment of collagen-related diseases such as liver cirrhosis.

Regarding the molecular interaction between Hsp47 and collagen, Dafforn *et al.* proposed a *flying capstan* model in which Hsp47 forms a closed “circlet” trimer trapping a (PPG)_10_ peptide on the side between α-helices hA and hG/hH in each subunit, which is composed of a core of three β-sheets surrounded by ten α-helices [9] (Figure S1). Subsequently, the consensus sequence on procollagen that is required for binding to Hsp47 was identified by a synthetic peptide approach. Koide *et al*. reported that triple helix formation is a prerequisite for the interaction of collagen-like model peptides with Hsp47 and that a Gly-Xaa-Arg sequence that is harbored in the triple helix structure serves as a binding motif for Hsp47 [10], [11], [12]. Moreover, cross-linking experiments have indicated that the amino-acid side chain at position −3 of collagen is directly recognized by a Hsp47 molecule, in addition to the critical arginine residue at position 0 [12]. However, very little information is currently available regarding the amino acid residues of Hsp47 that are involved in the interaction with collagen.

**Figure 1:**
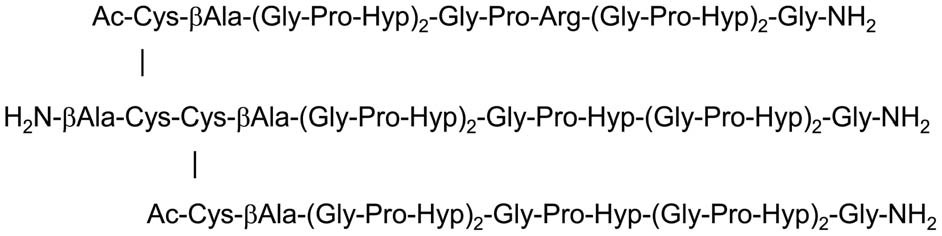
Structure of the synthetic trimeric collagen peptide. βAla and Hyp denote β-alanine and 4-hydroxyl-L-proline residues, respectively.

In the present study, we attempted to identify the collagen-binding site of Hsp47 with a structural approach. Hsp47 belongs to the serpin superfamily despite its lack of serine protease inhibitory activity [13]. This enabled us to build a 3D-structural model of Hsp47 with a homology modeling method, even though crystallographic data of Hsp47 itself has not been available to date. We performed nuclear magnetic resonance (NMR) spectroscopic analyses to characterize the interaction in solution between chicken Hsp47 and a synthetic collagen peptide trimer with a melting temperature of 44.5°C [14] (Figure 1). We observed that sophisticated multidimensional NMR approaches could not be applied because of the large molecular size, and more profoundly the aggregation-prone property of Hsp47. To address such difficulties, we used an amino acid selective-labeling approach in conjunction with site-directed mutagenesis, which together enable NMR spectral assignments of large proteins [15], [16], [17]. We successfully assigned the NMR peaks originating from selected amino acid residues of Hsp47 and mapped the collagen-binding site on the 3D structure of Hsp47 using the peaks as spectroscopic probes.

## Results and Discussion

In general, a solution with a high concentration is required for protein NMR measurements. We attempted to improve the solubility of human and chicken Hsp47 as recombinant proteins under a variety of solution conditions. Finally, we found that chicken Hsp47 could be soluble at a protein concentration of 0.2 mM in 20 mM HEPES buffer (pH 8.0) containing 150 mM NaCl, 15% (v/v) glycerol, and 0.02% (v/v) n-octyl-β-_D_-glucopyranoside, while the solubility of human Hsp47 never exceeded 0.04 mM under the solution conditions we tested, despite the similarity of their amino-acid sequences (almost 80% amino-acid identity) (Figure S1). However, even under the optimum solution conditions, a ^1^H-^15^N heteronuclear single-quantum correlation (HSQC) spectrum of uniformly ^15^N-labeled chicken Hsp47 exhibited severe peak broadening, which was probably due to its aggregation tendency (data not shown) and which hampered sequential assignment approaches that were based on multidimensional NMR spectra. In order to overcome these problems, we employed the amino-acid-specific labeling technique for the observation of NMR peaks originating from selected amino acid residues. We focused on the Hsp47 tryptophan and histidine residues because these amino acid residues are well conserved among species and distributed over the entire surface of the Hsp47 molecule. Therefore, these residues were expected to provide useful spectroscopic probes to characterize the interaction between Hsp47 and collagen.

**Figure 2:**
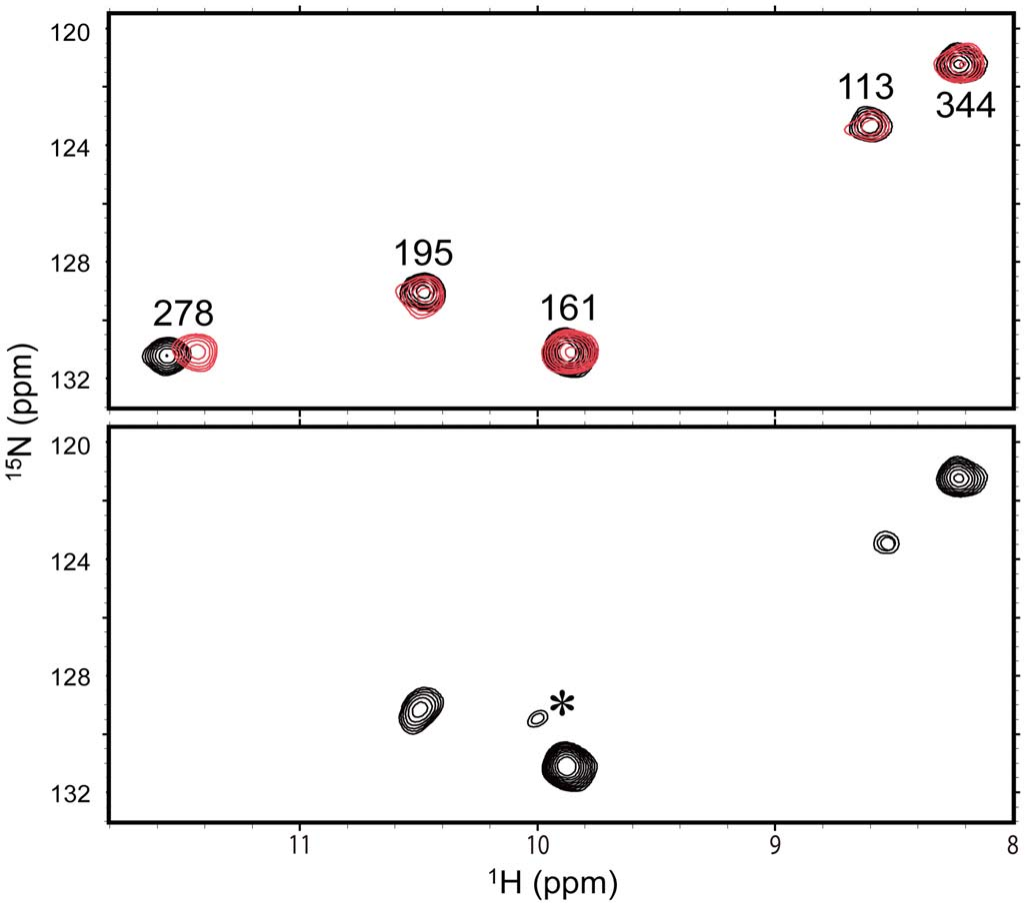
Probing collagen binding of Hsp47 using ^1^H-^15^N HSQC peaks originating from the tryptophan indole groups. ^1^H-^15^N HSQC spectra of the ε-imino groups of the tryptophan residues of wild-type (upper) and the W280Y mutant (lower) of Hsp47 in the absence (black) or presence (red) of trimeric collagen peptide. The assignment of the Trp280 peak was made by comparing the spectra of the wild-type and W280Y-mutated Hsp47. Asterisk indicates the peak originating from a denatured species arising during NMR measurement.

**Figure 3:**
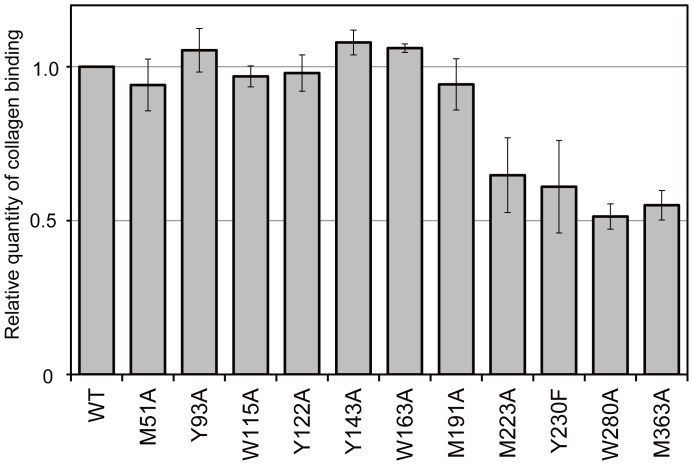
Mutational analysis of collagen binding of Hsp47. Wild-type and mutated Hsp47 recombinant proteins were mixed with affinity beads with or without immobilized collagen and visualized by western blotting using an anti-Hsp47 antibody. Relative quantities of the collagen-binding Hsp47 mutants are normalized against that of wild-type Hsp47. The values are the mean values ± S.D. of three independent experiments. Tyr230 was substituted with phenylalanine; alanine substitution resulted in protein denaturation.

Here, we describe our strategy by showing the procedures and the assignments of the ε-imino groups of the tryptophan residues and the interaction analyses using these peaks as probes. Chicken Hsp47 possesses five tryptophan residues at positions 115, 163, 197, 280, and 346. Figure 2 compares the ^1^H-^15^N HSQC spectra between wild-type and W280Y-mutated Hsp47 that were labeled with ^15^N at the ε-imino group. Among the five peaks observed in the wild-type spectrum, the peak exhibiting the proton resonance that is shifted most downfield is absent from the spectrum of the W280Y mutant, enabling us to assign the peak to Trp280. Likewise, the remaining peaks were all assigned by site-directed tyrosine substitutions (Figure S2). Upon addition of the trimeric collagen peptide, a chemical shift perturbation was observed for the Trp280 peak, while the remaining peaks were unaffected (Figure 2, Table S1). These results suggest that Trp280 is involved in the interaction with collagen. In fact, the binding assay using immobilized collagen confirmed that the affinity to collagen was significantly compromised by the alanine substitution of Trp280 (Figure 3).

Similarly, observable peaks originating from backbone-amide groups of histidine were assigned by amino acid-selective isotope labeling combined with site-directed mutagenesis (Figure S3). The chemical shift assignments are summarized in Table S1. By using these peaks, we probed the collagen-binding site of Hsp47 and observed chemical shift perturbations (His261 and His 373) and/or peak intensity attenuations (His202 and His225) upon addition of the trimeric collagen peptide.

**Figure 4:**
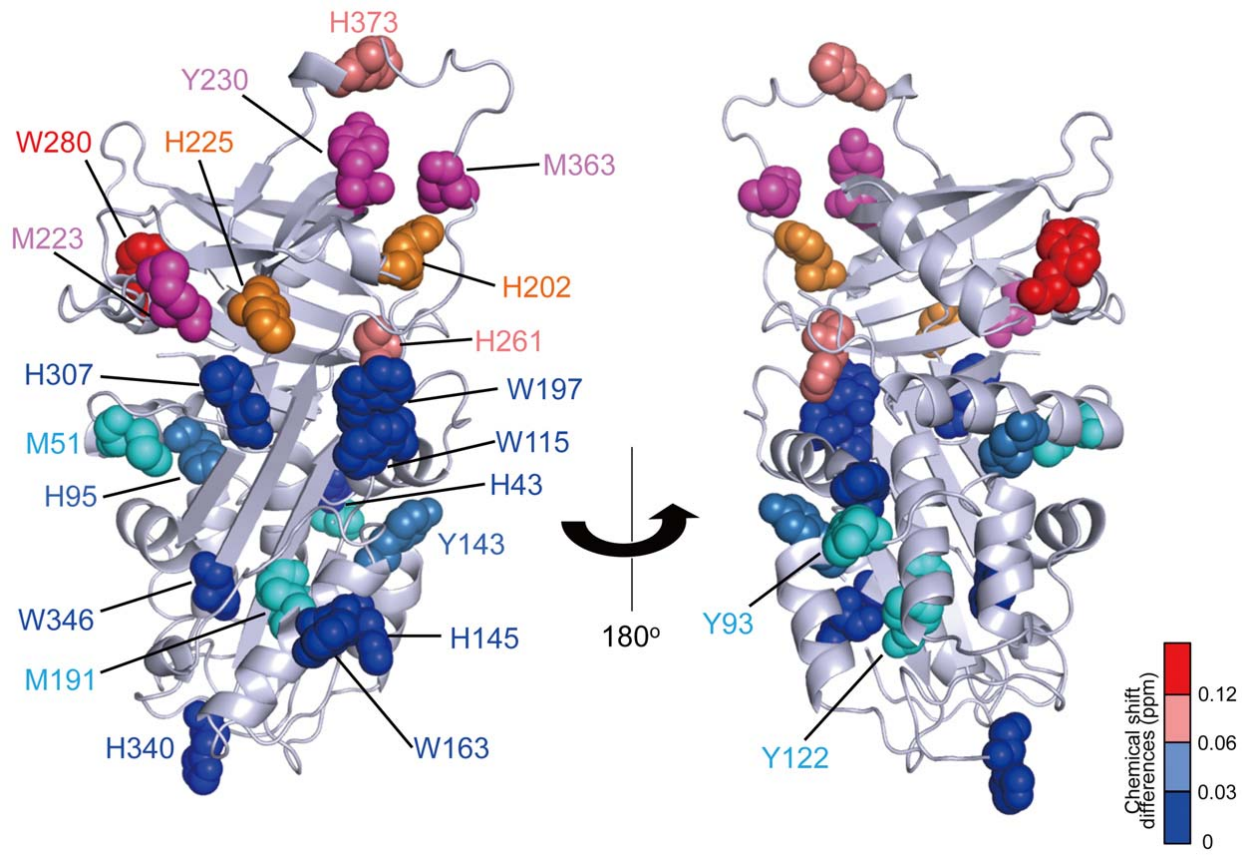
Mapping of the collagen-binding site of Hsp47 in the 3D-structural model. The tryptophan and histidine residues are mapped on the 3D-homology model of Hsp47 with a space-filling representation. Colors indicate the strength of the perturbation [(0.04Δ*δ*_N_^2^+Δ *δ*_H_^2^)^1/2^, where *δ*_N_ and *δ*_H_ represent the difference in nitrogen and proton chemical shifts, respectively] upon addition of the trimeric collagen peptide as follows: >0.12 ppm (red), 0.12 - 0.06 ppm (salmon pink), 0.06 - 0.03 ppm (sky blue), and <0.03 ppm (blue). The histidine residues whose peak intensity attenuations to an undetectable level upon addition of the peptide are shown in orange. The methionine and tyrosine residues are mapped with a space-filling representation according to the mutational effects on collagen-binding affinities observed as follows: >30% (magenta), and <10% (cyan) reductions upon mutations.

We mapped the amino acid residues whose NMR peaks were perturbed upon binding to the trimeric collagen peptide on the 3D structure of Hsp47 (Figure 4). Because the crystal structure of Hsp47 has yet to be elucidated, we made a 3D-structural model of chicken Hsp47 using the SWISS-MODEL homology-modeling server. The mapping indicated that the perturbed amino acid residues are located at the B/C β-barrel domain and the spatially proximal loop, which corresponds to the reactive center in serpin family proteins. Furthermore, the mutational analysis indicates that the amino acid substitutions in the B/C β-barrel domain and the loop (M223A, Y230F, W280A, and M363A) significantly compromise the interaction with immobilized collagen, while the mutations in the α/β domain (M51A, Y93A, Y122A, Y143A, and M191A) had no impact (Figure 3, Figure 4, Figure S1). The CD data confirmed that all these mutants retained structural integrity (Figure S4), indicating that Met223, Met363, and Tyr230 are involved in the interaction.

Based on these data, we conclude that Hsp47 binds collagen through the B/C β-barrel domain and a nearby loop. Most of the residues thus identified in the collagen binding site of Hsp47 are conserved across species (Figure S1). The widespread spatial distributions of the perturbed residues suggest that Hsp47 undergoes a conformational change upon binding to collagen because the binding site includes the loop with structural plasticity [9]. Previously reported cross-linking data suggested that the longer axis of Hsp47 is oriented along the helical axis of the collagen triple helix in the complex [12]. On the other hand, in the flying capstan model, the collagen-binding site is speculated to consist of α-helices hA and hG/hH [9]. Such a binding mode is apparently incompatible with the present data. Indeed, amino acid substitutions (H43 and M51) in hA helix have no impact on collagen binding (Figure 4, Figure S1). One possible explanation for this discrepancy is that the cross-linking experiment traps transiently interacting species, such as the encounter complex formed between Hsp47 and collagen, whilst the present NMR data identify the collagen-accommodating site on Hsp47. The electrostatic surface potential of Hsp47 indicates that there exist negative charge clusters in the B/C β-barrel domain as well as one side of the α/β domain (Figure S5). It is possible that these negatively charged surfaces interact with the arginine residues in collagen.

It has recently been reported that collagen secretion and accumulation in the liver during the progression of cirrhosis are dramatically suppressed by Hsp47 knockdown using siRNA packaged in vitamin A-coupled liposomes, which preferentially targeted hepatic stellate cells [18]. Based on the present NMR data, further studies using collagen analogs labeled with stable isotopes or paramagnetic probes could be employed to build a more detailed docking model between Hsp47 and collagen. Such detailed structural information would offers an opportunity to design small molecule inhibitors of the interaction between Hsp47 and procollagen that may be effective in the treatment of fibrotic diseases by preventing collagen secretion into the extracellular spaces.

## Materials and Methods

### Preparation of Hsp47 Variants by Site-directed Mutagenesis

Site-directed mutagenesis was conducted to produce a series of Hsp47 mutants: W115Y, W115A, W163Y, W163A, W197Y, W197A, W280Y, W280A, W346Y, W346A, H43A, H43Y, H95Y, H95A, H95Q, H140Y, H145Y, H196Y, H202Y, H203Y, H203A, H225Y, H225A, H261Y, H261A, H249Y, H261Q, H302Y, H307Y, H340Y, H340A, H340Q, H373Y, H373A, H373Q, M51A, M191A, M205A, M223A, M258A, M363A, Y42F, Y93A, Y122A, Y141A, Y143A, Y230A, Y230F, Y353F, Y358F, F201Y, F211Y, F256Y, and F389Y. The DNA fragment encoding amino acid residues of Hsp47 was cloned into the pET-21a vector (Novagen) with an N-terminal polyhistidine tag moiety. The pET-21a vector carrying wild-type chicken Hsp47 was used as a template for site-directed mutagenesis. PCR was performed with two synthetic oligonucleotide primers containing the desired mutation. PCR products were digested with DpnI and subsequently transformed into the *Escherichia coli* strain DH5α. The mutations were confirmed by DNA sequencing using an ABI 3100*x* genetic analyzer.

### Protein Expression and Purification of Wild-type and Mutated Hsp47 Proteins

Wild-type and mutated Hsp47 recombinant proteins were individually expressed in the *Escherichia coli* BL21(DE3)CodonPlus strain (Stratagene) in LB medium or M9 medium with [^15^N]NH_4_Cl, L-[indole-^15^N]tryptophan, or L-[α-^15^N]histidine using previously described protocols [16], [19]. Production of recombinant proteins was induced by the addition of 0.1 mM isopropyl β-_D_-thiogalactopyranoside. The polyhistidine fusion protein was purified from cell lysates with a DEAE column and then a Ni^2+^-nitrilotriacetic acid column (GE Healthcare). The fusion protein was cleaved by incubation with Factor Xa protease (Novagen), and the polyhistidine tag was removed by a Ni^2+^-nitrilotriacetic acid column. CD spectra of wild-type and mutated Hsp47 proteins were measured at 30°C on a Jasco J-720WI apparatus, using a 1.0-mm path length quarts cell. Many of these mutated proteins formed inclusion bodies in *E. coli* (W197A, H95Y, H95A, H203Y, H261A, H340Y, H340A, H373Y, H373A, H373Q, Y42F, Y141A, Y353F, Y358F, M205A, M258A, F201Y, F211Y and F389Y) or exhibited impaired folding (H43Y, H225Y, H261Y, Y230A and F256Y), although the structural integrity of the remaining mutants was confirmed by CD and NMR measurements (Figure 2, Figure S2, S3 and S4).

### Preparation of Peptide

Details of the construction and purification of the trimeric collagen peptide have been described previously [14]. Briefly, the heterotrimeric collagen peptide was synthesized by combination of the standard solid-phase method and subsequent regioselective disulfide-bond formation.

### NMR Measurements

The wild-type or mutated chicken Hsp47 with isotope labeling was dissolved at a concentration of 0.2 mM in 20 mM HEPES buffer (pH 8.0) containing 150 mM NaCl, 15% (v/v) glycerol, 0.02% (v/v) n-octyl-β-_D_-glucopyranoside, and 10% (v/v) ^2^H_2_O in the presence or absence of 0.2 mM trimeric collagen peptide. NMR spectral measurements were made on a Bruker DMX-500 spectrometer that was equipped with a cryogenic probe, which was critical for minimizing the denatured species arising during NMR measurement. The probe temperature was set to 30°C, where the trimeric collagen peptide had been confirmed to maintain a triple helix structure [14].

### 3D-structural Model

The crystal structure at 2.09-Å resolution of neuroserpin, which is a member of the serpin superfamily (PDP code: 3FGQ) [20], was used as a template for the tertiary structure of chicken Hsp47. The SWISS-MODEL homology-modeling server (http://swissmodel.expasy.org/) was used to produce the homology coordinate, and PyMOL (http://www.pymol.org/) was employed to view the output produced by the homology server. Electrostatic surface potential was calculated by the apbs plugin in PyMOL [21]. PDB data was converted to PQR format by PDB2PQR [22].

### Collagen-binding Assay

Porcine type-I collagen (Nitta Gelatin Inc.) was immobilized onto cyanogen bromide-activated Sepharose 4B (GE Healthcare). Immobilized collagen was kept at 4°C in 50 mM HEPES buffer (pH 8.0) containing 150 mM NaCl and 15% (v/v) glycerol. Each purified Hsp47 or its mutants (300 µL) was mixed with the binding buffer [50 mM HEPES (pH 8.0), 150 mM NaCl, 20 mM imidazole, 15% (v/v) glycerol, and 0.02% (v/v) n-octyl-β-_D_-glucopyranoside] and a 50-µL bed of the affinity beads with or without immobilized collagens. The binding reaction was carried out at 4°C for 2 h with gentle mixing. After washing the beads twice with the binding buffer, proteins retained on the beads were eluted by adding 2 × sodium dodecyl sulfate sample buffer [125 mM Tris-HCl (pH 6.8), 10% (v/v) Glycerol, 2% (w/v) sodium dodecyl sulfate, 0.04% (w/v) Bromophenol Blue, and 5% (v/v) 2-mercaptoethanol], incubated at 95°C for 3 min, and then separated by SDS-PAGE on a 10% gel. Proteins were transferred from a polyacrylamide gel to a nitrocellulose membrane and then visualized by western blotting using an anti-Hsp47 antibody (SPA470, Stressgen, Victoria, Canada). For the densitometric quantification of the proteins, the software application ImageJ (http://rsbweb.nih.gov/ij/) was used.

## Supporting Information

Figure S1 **Sequence conservation and secondary/tertiary structural elements of Hsp47.** Alignment was generated by ClustalW. Secondary and tertiary structures of 3D structural model of chicken Hsp47 are shown above its primary structure; the α-helix is depicted by a cylinder, β-strand by an arrow (A-sheet; green, B-sheet; purple, and C-sheet; yellow), and loop region by dashed line. The key residues are highlighted in white letters on colored backgrounds (in the same colors as those in Figure 4) with residue numbers.
(PDF)

Figure S2 **^1^H-^15^N HSQC spectral assignments of tryptophan indole peaks of Hsp47 by site-directed mutagenesis.** ^1^H-^15^N HSQC peaks originating from the ε-imino groups of tryptophan residues are compared between the wild-type and tryptophan-to-tyrosine mutants of Hsp47. Asterisk indicates the peak originating from denatured species arising during NMR measurement.
(PDF)

Figure S3 **^1^H-^15^N HSQC spectral assignments of histidine peaks of Hsp47 by site-directed mutagenesis.** ^1^H-^15^N HSQC peaks originating from the backbone amide groups of histidine residues are compared between the wild-type and mutants of Hsp47 in the absence (black) or presence (red) of trimeric collagen peptide.
(PDF)

Figure S4 **CD spectra of Hsp47 mutants used for the collagen-binding assay.** CD data confirmed the structural integrity of Hsp47 mutants. All proteins were dissolved at a concentration of 5.0 µM in 10 mM sodium phosphate buffer (pH 7.5) containing 150 mM NaCl. Four scans were averaged for each sample.
(PDF)

Figure S5 **Electrostatic surface potential of Hsp47.** The surface potential from −6 kT in red to +6 kT in blue is mapped on the 3D-homology model of Hsp47 with a surface representation.
(PDF)

Table S1 **Chemical shifts of the Hsp47 peaks used as spectroscopic probes in the absence or presence of the trimeric collagen peptide.**
(PDF)
