# Supplementary figures and images for: NMR and Mutational Identification of the Collagen-Binding Site of the Chaperone Hsp47
Source: PLoS One. 2012 Sep 25;7(9):e45930. doi: 10.1371/journal.pone.0045930 (PMC3457968; doi:10.1371/journal.pone.0045930)

# Figure S1

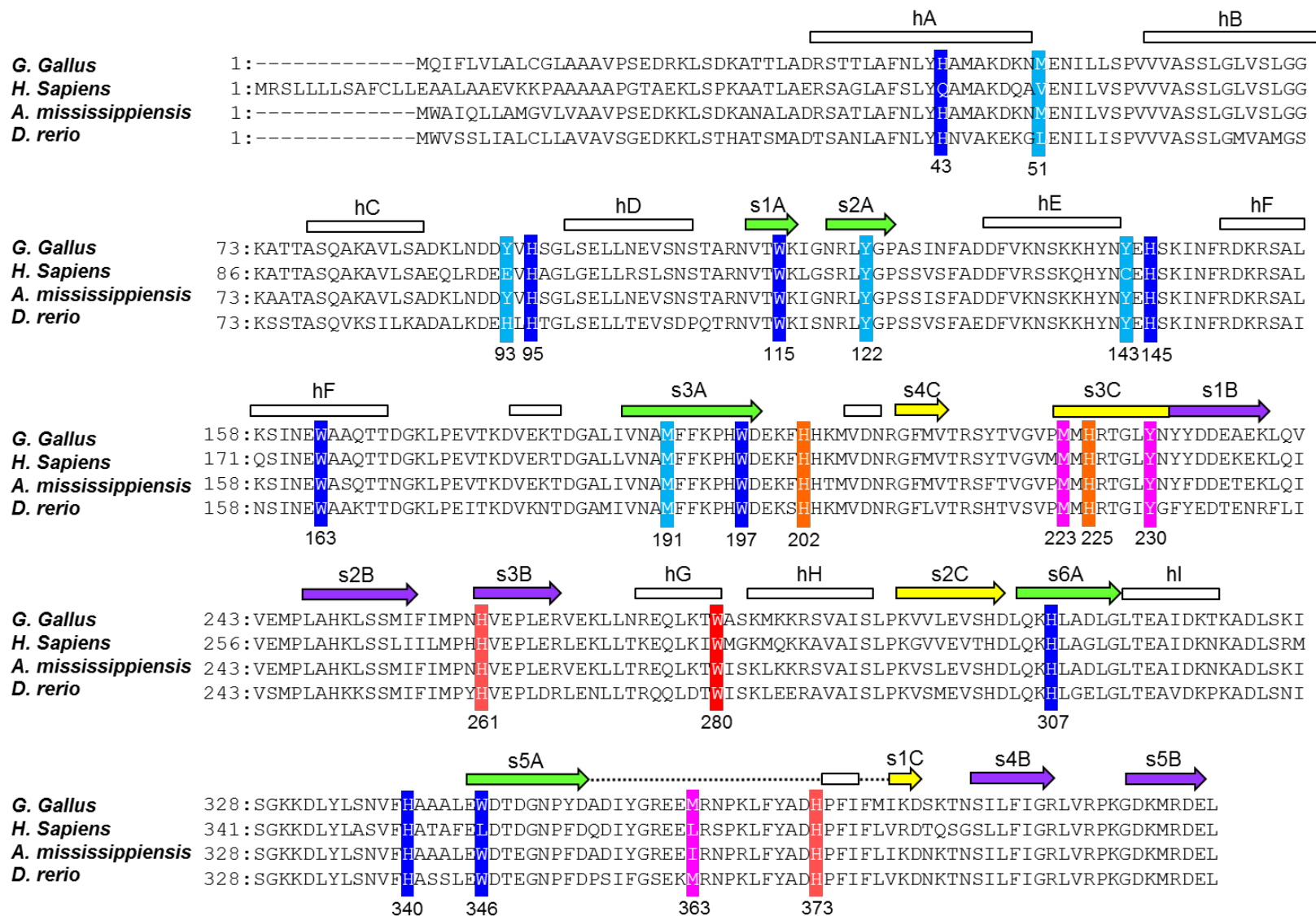

Supplement: Figure S1 — Sequence conservation and secondary/tertiary structural elements of Hsp47. Alignment was generated by ClustalW. Secondary and tertiary structures of 3D structural model of chicken Hsp47 are shown above its primary structure; the α-helix is depicted by a cylinder, β-strand by an arrow (A-sheet; green, B-sheet; purple, and C-sheet; yellow), and loop region by dashed line. The key residues are highlighted in white letters on colored backgrounds (in the same colors as those in Figure 4) with residue numbers. (PDF) [file pone.0045930.s001.pdf]

Figure S2

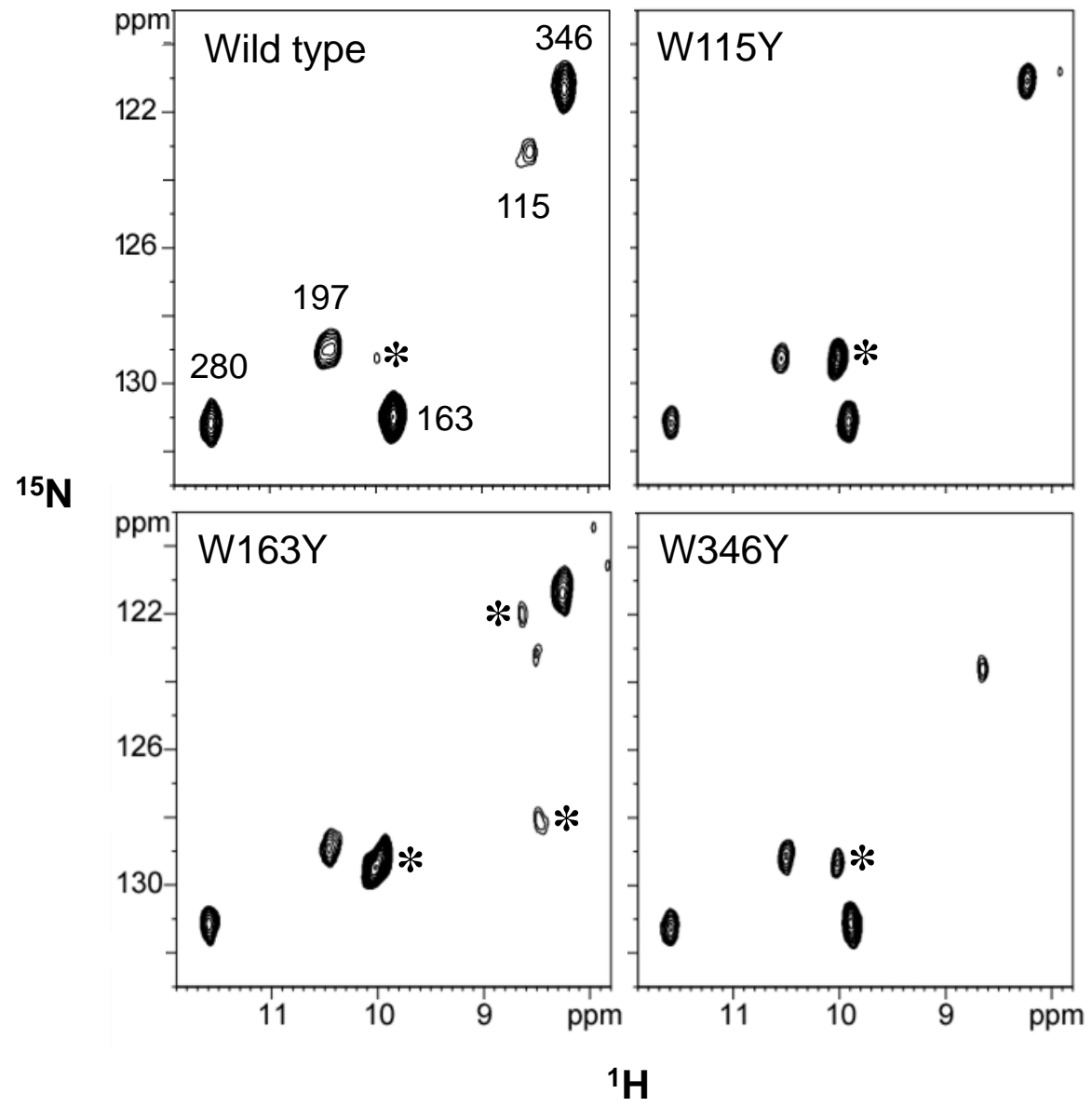

Supplement: Figure S2 — 1H-15N HSQC spectral assignments of tryptophan indole peaks of Hsp47 by site-directed mutagenesis. 1H-15N HSQC peaks originating from the ε-imino groups of tryptophan residues are compared between the wild-type and tryptophan-to-tyrosine mutants of Hsp47. Asterisk indicates the peak originating from denatured species arising during NMR measurement. (PDF) [file pone.0045930.s002.pdf]

**Figure S3**

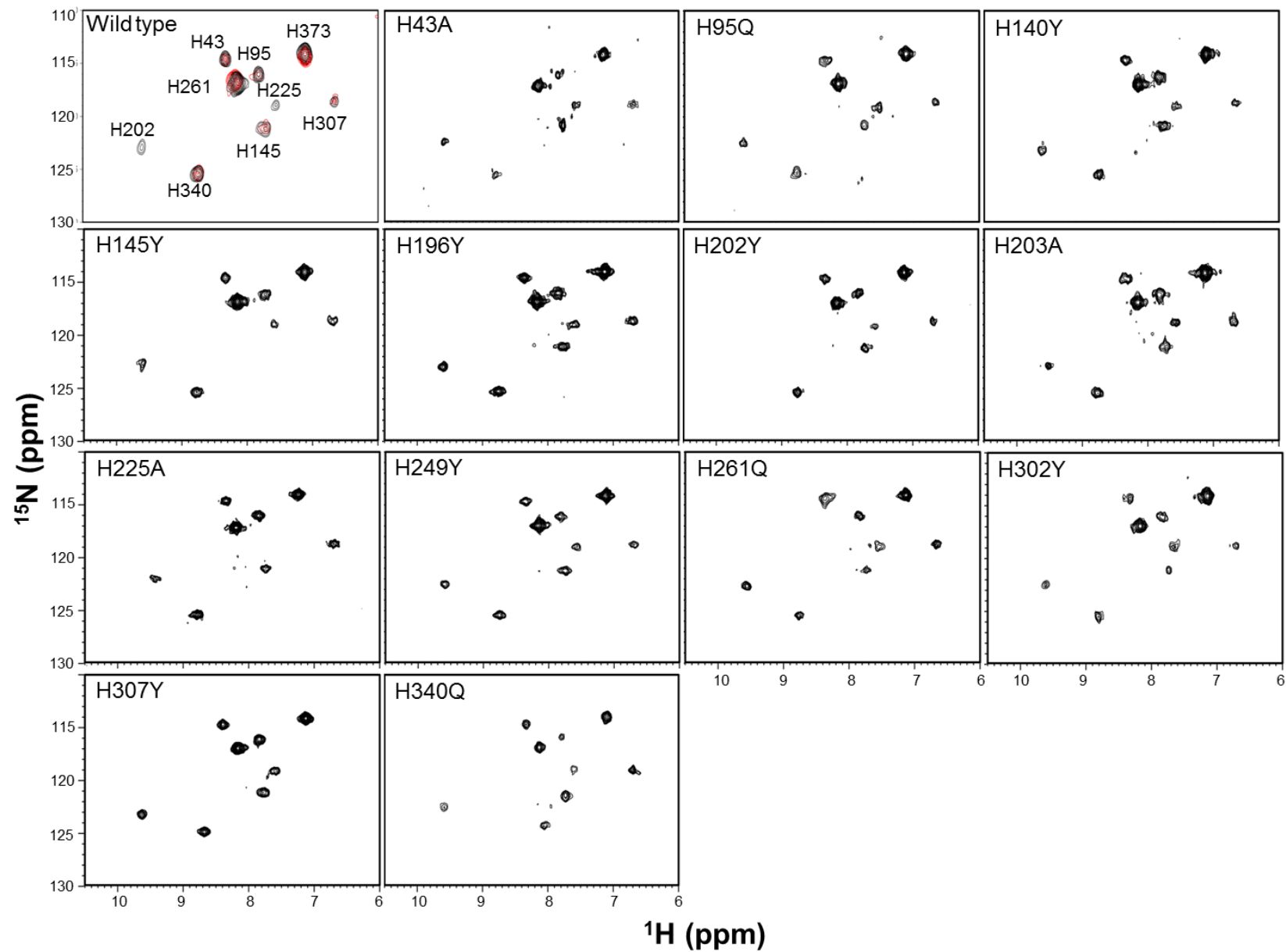

Supplement: Figure S3 — 1H-15N HSQC spectral assignments of histidine peaks of Hsp47 by site-directed mutagenesis. 1H-15N HSQC peaks originating from the backbone amide groups of histidine residues are compared between the wild-type and mutants of Hsp47 in the absence (black) or presence (red) of trimeric collagen peptide. (PDF) [file pone.0045930.s003.pdf]

**Figure S4**

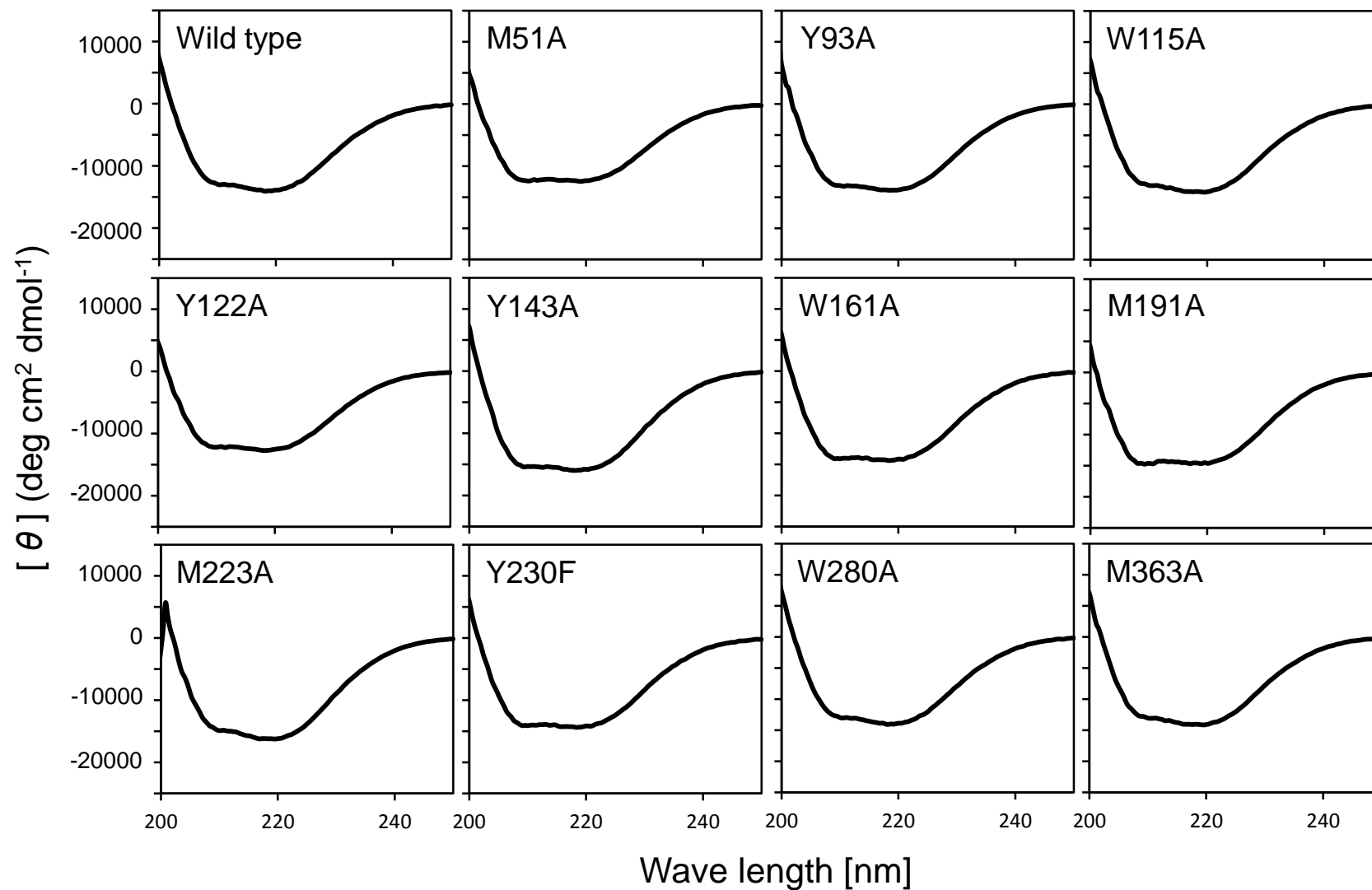

Supplement: Figure S4 — CD spectra of Hsp47 mutants used for the collagen-binding assay. CD data confirmed the structural integrity of Hsp47 mutants. All proteins were dissolved at a concentration of 5.0 µM in 10 mM sodium phosphate buffer (pH 7.5) containing 150 mM NaCl. Four scans were averaged for each sample. (PDF) [file pone.0045930.s004.pdf]

Figure S5

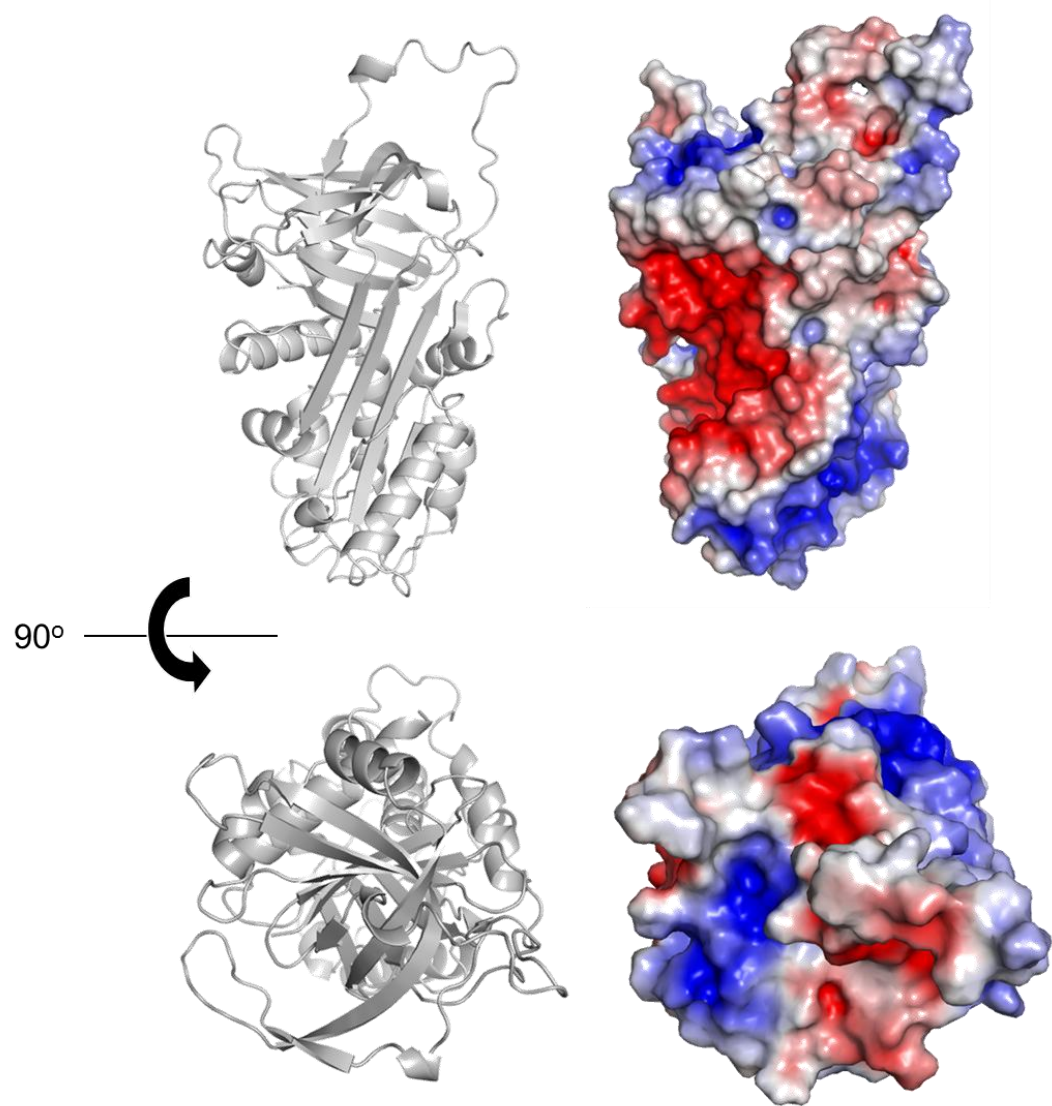

Supplement: Figure S5 — Electrostatic surface potential of Hsp47. The surface potential from −6 kT in red to +6 kT in blue is mapped on the 3D-homology model of Hsp47 with a surface representation. (PDF) [file pone.0045930.s005.pdf]
