# Supplementary material for: NMR and Mutational Identification of the Collagen-Binding Site of the Chaperone Hsp47
Source: PLoS One. 2012 Sep 25;7(9):e45930. doi: 10.1371/journal.pone.0045930 (PMC3457968; doi:10.1371/journal.pone.0045930)
Supplement: Table S1 — Chemical shifts of the Hsp47 peaks used as spectroscopic probes in the absence or presence of the trimeric collagen peptide. (PDF) [file pone.0045930.s006.pdf]

Table S1

Residue	Trimeric collagen peptide			
	(-)		(+)	
**Trp**	**ε-N**	**ε-NH**	**ε-N**	**ε-NH**
	(ppm)		(ppm)	
115	123.3	8.6	123.4	8.6
163	131.1	9.9	131.1	9.9
197	129.0	10.5	129.1	10.5
280	131.2	11.6	131.1	11.4
346	121.2	8.2	121.2	8.2
**His**	**α-N**	**α-NH**	**α-N**	**α-NH**
	(ppm)		(ppm)	
43	114.6	8.3	114.7	8.3
95	116.0	7.8	116.1	7.8
140	n.d.[b]	n.d.	n.d.	n.d.
145	121.2	7.7	121.0	7.7
196	n.d.	n.d.	n.d.	n.d.
202	122.9	9.6	n.d.	n.d.
203	n.d.	n.d.	n.d.	n.d.
225	118.9	7.6	n.d.	n.d.
249	n.d.	n.d.	n.d.	n.d.
261	116.9	8.1	116.7	8.2
302	n.d.	n.d.	n.d.	n.d.
307	118.6	6.7	118.5	6.7
340	125.4	8.7	125.4	8.7
373	114.1	7.1	114.4	7.1

[b]not detectable.
